# Belgian National Survey on Tinea Capitis: Epidemiological Considerations and Highlight of Terbinafine-Resistant *T. mentagrophytes* with a Mutation on SQLE Gene

**DOI:** 10.3390/jof6040195

**Published:** 2020-09-29

**Authors:** Rosalie Sacheli, Saadia Harag, Florence Dehavay, Séverine Evrard, Danielle Rousseaux, Akole Adjetey, Laurence Seidel, Kim Laffineur, Katrien Lagrou, Marie-Pierre Hayette

**Affiliations:** 1 Department of Clinical Microbiology, Belgian National Reference Center for Mycoses, University Hospital of Liege, 4000 Liège, Belgium; aadjetey@chuliege.be (A.A.); mphayette@chuliege.be (M.-P.H.); 2 Department of Dermatology, University Hospital St Pierre Brussels, 1000 Brussels, Belgium; Saadia_HARAG@stpierre-bru.be (S.H.); Florence.Dehavay@ulb.be (F.D.); 3 Department of Clinical Microbiology, CHR, Citadelle, Regional Hospital of Liege, 4000 Liège, Belgium; Severine.Evrard@chrcitadelle.be; 4 Department of Clinical Microbiology, CHC Group, Montlegia Hospital, 4000 Liege, Belgium; Danielle.rousseaux@chc.be; 5 Department of Biostatistics, University Hospital of Liege, 4000 Liège, Belgium; laurence.seidel@chuliege.be; 6 Department of Microbiology, St Luc Hospital Bouge, 5004 Bouge, Belgium; k.laffineur@labstluc.be; 7 Laboratory of Clinical Bacteriology and Mycology, Belgian National Reference Center, University Hospital of Leuven, 3000 Leuven, Belgium; katrien.lagrou@uzleuven.be

**Keywords:** tinea capitis, dermatophytes, Belgium, resistance, terbinafine, prevalence

## Abstract

Background: In this last decade, a huge increase in African anthropophilic strains causing tinea capitis has been observed in Europe. The Belgian National Reference Center for Mycosis (NRC) conducted a surveillance study on tinea capitis in 2018 to learn the profile of circulating dermatophytes. Methods: Belgian laboratories were invited to send all dermatophyte strains isolated from the scalp with epidemiological information. Strain identification was confirmed by ITS (Internal Transcribed Spacer) sequencing. Mutation in the squalene epoxidase (SQLE) gene was screened by PCR. Results: The main population affected by tinea capitis was children from 5–9 years. Males were more affected than females. The majority of the strains were collected in the Brussels area followed by the Liege area. Among known ethnic origins, African people were more affected by tinea capitis than European people. The major aetiological agent was *Microsporum audouinii*, followed by *Trichophyton soudanense*. One strain of *Trichophyton mentagrophytes* has been characterized to have a mutation on the squalene epoxidase gene and to be resistant to terbinafine. Conclusions: African anthropophilic dermatophytes are mainly responsible for tinea capitis in Belgium. People of African origin are most affected by tinea capitis. The monitoring of terbinafine resistance among dermatophytes seems necessary as we have demonstrated the emergence of resistance in *T. mentagrophytes*.

## 1. Introduction

Tinea capitis is a dermatophytic infection of the hair and scalp often associated with signs of inflammation and alopecia and, more rarely, giving rise to kerion or favus. This disease is common in children and rarer in adults, but its prevalence in the population can reach 40% in some communities. It is known to be highly contagious among family members and among school children [1,2]. The epidemiology of tinea capitis varies within different geographical areas and is dependent on factors such as climate, temperature, relative humidity, and precipitation of the concerned regions. In recent decades, the epidemiology of tinea capitis in Europe and around the world has changed, and an increase in the incidence of this pathology has been noticed. Changes concern especially the relative prevalence of different aetiological agents. Indeed, a shift in incidence from anthropophilic to zoophilic dermatophytes was noticed in many countries after 1970, with *Microsporum canis* being predominant in many parts of Europe [3,4,5,6,7]. Immigration waves, multiple travels of the population, changes in hygiene habits, and the use of griseofulvin against *Microsporum audouinii* have contributed to these related modifications. However, in urban areas, especially this last decade, anthropophilic strains remain predominant in cities such as Paris, Birmingham, and some regions of the USA [8,9,10,11,12,13,14]. An understanding of changing epidemiology and prevalence of causative organisms is important in deciding appropriate therapy for tinea capitis, but this has been poorly explored in Belgium. For this reason, and due to the lack of recent reports concerning tinea capitis in Belgium, the National Reference Center for Mycoses decided to organise a national survey to collect information concerning the status of tinea capitis in Belgium and to evaluate the aetiological agents responsible for this pathology. The aim of this study was also to evaluate the impact of immigration waves and other potential factors in the recrudescence of some African anthropophilic species in our regions, such as *M. audouinii* or *T. soudanense*. The present study reports the distinctive characteristics of the aetiological agents of tinea capitis in Belgium and the epidemiological features of the reported cases.

## 2. Materials and Methods

### 2.1. Design and Data Collection

The participation of fourteen laboratories around Belgium was requested for this national study. The collaboration of dermatologists from the hospitals was required to fill a form for every case of tinea capitis. Gender, age, site of involvement, ethnic origin, geographical area, information about familial cases, the possession of an animal, and acquired infection in school or kindergarten were asked for each subject. This information was unfortunately not obtained for each patient. In total, 337 cases of tinea capitis were recorded from 1 January 2018 to 31 December 2018.

### 2.2. ITS PCR and Sequencing

All 337strains were identified by DNA sequencing as a reference method for species confirmation. The rRNA ITS2 region was sequenced for all strains. Briefly, DNA was extracted from a mycelial mass (cultured in liquid Sabouraud dextrose broth) using the Maxwell SEV 16 cell kit (Promega, Madison, WI, USA) with the following pretreatment. Briefly, after growth, the liquid culture was centrifuged at 3000× *g* for 10 min; the pellet culture was immersed into 1 mL of water placed into tubes with 0.5 mm microbeads (Omni international, Tulsa, OK, USA). Samples were vortexed for 30 min. Then, the liquid was removed, and a chemical lysis was done with 20 µL Proteinase K on 200 µL ATL buffer and 180 µL AL buffer (all three manufactured by Qiagen, Hilden, Germany). Following this pretreatment, DNA was extracted with the Maxwell^®^ (Promega, Madison, WI, USA) instrument following the manufacturer’s instructions. The ITS2 region was amplified using the ITS86 forward primer 5′GTG-AAT-CAT-CGA-ATC-TTT-GAA 3′ and ITS4 reverse primer 5′TCC-TCC-GCT-TAT-TGA-TAT-GC 3′ [15]. Purification of PCR products was then performed using the Exosap IT technique (Amersham, GE Healthcare Europe GmbH, Machelen, Belgium). Amplification was performed on a classical thermocycler (Thermohybaid, ThermoScientific, Merelbeke, Belgium). Bidirectional sequence data were generated after purification using the BigDye terminator sequencing kit (Applied Biosystems, Life technologies, Merelbeke, Belgium). Sequenced products were finally purified using the kit clean Seq Agencourt (Beckman Coulter Life Science, Marseille, France). The sequencing was done on the automated ABI 3500/3500XL (Applied Biosystem, Life technologies, Merelbeke, Belgium). Sequences were edited using the ABI Sequence Scanner V.1.0 software (Applied Biosystems, Life Technologies, Merelbeke, Belgium). Sequences generated by the software were then compared to the CBS (Centraal Bureau voor Schimmelcultures, Utrecht, The Netherland) database, which comprises several databases including GenBank.

### 2.3. Dermagenius^®^ Real-Time Multiplex PCR

If no identification was obtained by ITS sequencing, as was the case for 11 strains, a DermaGenius^®^ real-time multiplex PCR (PathoNostics, Maastricht, The Netherlands) was applied. Extraction was performed on positive cultures. A piece of culture was scrapped from the Sabouraud medium and immersed into 475 µL of ATL buffer (Qiagen, Hilden, Germany). Twenty-five microliters of proteinase K was added to each tube. Incubation at 56 °C overnight was done under agitation at 900 rpm. After this pretreatment, DNA extraction was done using the QIAamp DNA minikit (Qiagen, Hilden, Germany) following the manufacturer’s instructions. The PCR was also performed according to manufacturer’s instructions: 5 μL of extracted DNA was added to the PCR mix, and a LightCycler 480 II (Roche, Bâle, Switzerland) was used for amplification and melting curve analysis. Data analysis was done using the 2nd-derivative and Tm-calling function of the LC480 software (version 1.5.1.62 SP2).

### 2.4. Antifungal Susceptibility Testing

Determination of minimal inhibitory concentration (MIC) was done according to the EUCAST E.Def 9.3.1 procedure with modifications described by Arendrup et al. [16]. Inoculum suspensions were prepared, filtered through a sterile filter with a pore diameter of 11 µm (Merck, Darmstadt, Germany) to remove hyphae, and diluted 1:10 with sterile distilled water to obtain a final inoculum of 2–5 × 10^5^ CFU/mL as described in E.Def 9.3.1. Suspension was supplemented with chloramphenicol 2 µL/mL of a stock solution of 50 mg/mL (Merck, Darmstadt, Germany) and cycloheximide, 6 µL/mL of a stock solution of 100 mg/mL (Merck, Darmstadt, Germany). The final concentration range for terbinafine, voriconazole, itraconazole, and amorolfine (after inoculation) was 0.008–8 mg/L. The reading of the MIC50 value (MIC where 50% of microorganisms are inhibited) was done according to the described recommendations using visual and an automated reading at 490 nm with a Multiscan FC spectrophotometer (Thermo Scientific, Waltham, MA, USA) [16].

### 2.5. Squalene Epoxidase (SQLE) Gene Analysis

Total DNA was extracted from fresh fungal cultures growing in liquid Sabouraud medium (Merck, Darmstadt, Germany) using the Maxwell 16 Cell SEV kit (Promega, USA). The PCR amplifying the SQLE gene was done as described by Yamada et al. [17]. Purification of PCR products was then performed using the Exosap IT technique (Amersham, GE Healthcare Europe GmbH, Belgium). Bidirectional sequence data were generated after purification using the BigDye terminator sequencing kit (Applied Biosystems, Life Technologies, Merelbeke, Belgium). Sequenced products were finally purified using the kit clean Seq Agencourt (Beckman Coulter Life Science, Villepinte, France). The sequencing was done on the automate ABI 3500/3500XL (Applied Biosystems, Life Technologies, Belgium). Sequences were edited using the ABI Sequence Scanner V.1.0 software (Applied Biosystems, Life Technologies, Merelbeke, Belgium). Forward and reverse sequences generated by the software were then blasted, and the consensus alignment was transduced in Expasy portal (SIB Bioinformatics Resource Portal, Lausanne, Switzerland) on amino acid sequence. The delivery sequence was then compared to the reference SQLE amino-acid sequence of *T. mentagrophytes* GenBank: BAL48859.1.

### 2.6. Statistical Analysis

For the statistical analysis, the software SAS was used (version 9.4). To compare the different variables, a Chi-square test was applied. Statistical significance was defined for *p*-value < 0.05.

### 2.7. Ethical Agreement

The design of the study was submitted to the ethical committee of our institution, and conditions of the study have been accepted; reference N° 2018/36.

## 3. Results

### 3.1. Epidemiological Considerations

In total, 14 laboratories took part in the study. We collected data from a total of 337 patients with alopecic plaques highly suggestive of the diagnosis of tinea capitis and presenting a positive culture for dermatophytes. Information regarding the included cases is presented in Table 1. The majority of samples were collected in the Brussels area, followed by the Liege area. Prevalence of tinea capitis was higher among males as dermatophytes infections concerns 214 males (63.5%) and 123 females (36.5%) (*p*-value < 0.0001). Children under 10 were more susceptible to developing a tinea capitis due to dermatophytes, as among the 337 samples, 62 (18.2%) were >10 years (*p*-value < 0.0001). Among children under ten, the age group 5–9 years (*n* = 165, 49%) was more susceptible to dermatophyte infections than the age group 0–4 years (*n* = 110, 32.6%). The age range was 0 to 57 years old with an average of 8.03 years.

**Figure 1:**
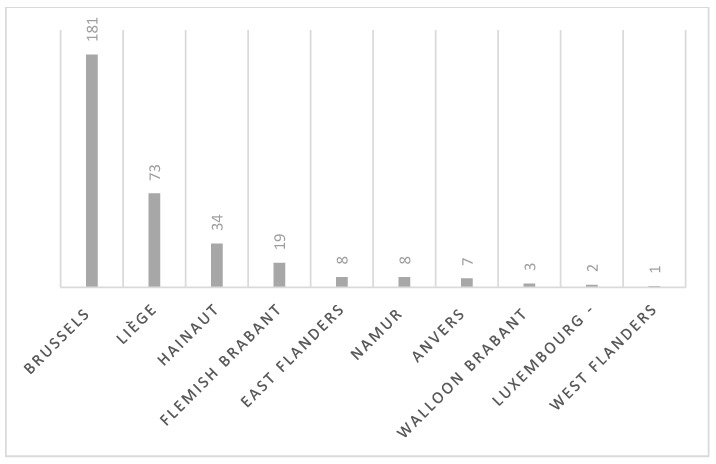
Geographical distribution all around Belgium of cases of tinea capitis collected during the study (*n* = 336, one localization not known).

Regarding the geographical area, the information was obtained for 336/337 patients as one patient was homeless. The majority of the cases were from the Brussels area (*p*-value < 0.0001). Indeed among 336 cases, 181 cases (53.87%) were from this area. Seventy-three cases (21.7%) were from Liege, 34 (10.10%) were from Hainaut, 19 (5.65%) were from Flemish Brabant, 8 (2.4%) were from East Flanders, 8 (2.4%) were from Namur, 7 (2.1%) were from Anvers, 3 (0.9%) were from Walloon Brabant, 2 (0.6%) were from Luxembourg, and 1 case was from West Flanders (0.3%) (Figure 1).

The ethnic origin was also asked for each patient. Only 119/337 answers were obtained for this variable. Among available results, we can clearly see that the majority of the patients were of African origin (112/119, 94.1%, *p*-value < 0.0001). Among these, 26 (21.8%) were from Guinea, 14 were from Congo and Cameroon (11.8%), 11 were from Morocco (9.2%), and 10 were from the Ivory Coast (8.4%). The other countries, concerning less than ten cases, are listed in Table 2.

**Figure 2:**
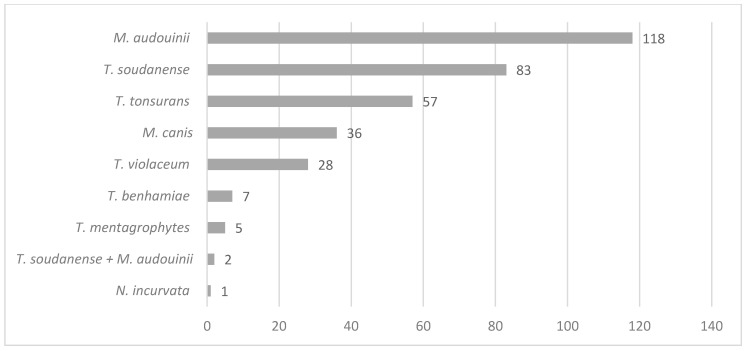
Description of the prevalence of dermatophytes species among the 337 cases of recorded tinea capitis.

Regarding the aetiological agents responsible for tinea capitis in Belgium, Figure 2 shows the repartition of dermatophytes circulating in Belgium. The main agent is *M. audouinii,* concerning 118/337 cases (35%, *p*-value < 0.0001), followed by *Trichophyton soudanense* with 83/337 cases (24.6%), then *Trichophyton tonsurans* (57/337, 16.9%), *Microsporum canis* (36/337, 10.7%), *Trichophyton violaceum* (28/337, 8.3%) *Trichophyton benhamiae* (7/337, 2.1%), *Trichophyton mentagrophytes* (5/337, 1.2%), *Nannizzia incurvata* (1/337, 0.3). Two mixed infections by *T. soudanense* and *M. audouinii* concomitantly were also recorded (0.6%).

**Figure 3:**
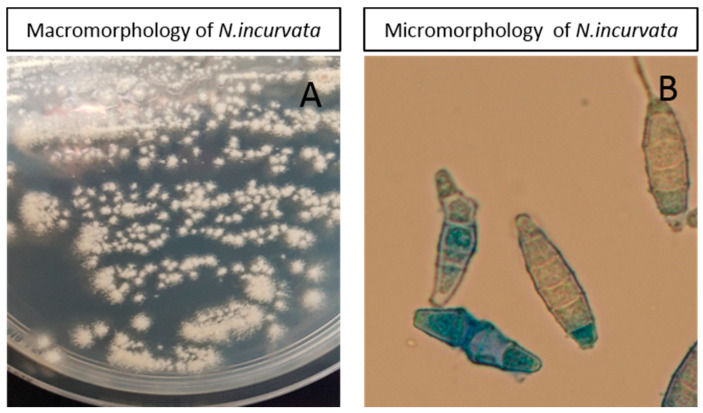
Macromorphology (**A**) and micromorphology (**B**) of the *N. incurvata* strain isolated in the study.

*Nannizia incurvata,* like the other strains of the study, was characterised by ITS–PCR but also confirmed by culture and microscopy (Figure 3). Culture was flat, powdery in texture, and beige-buff in color with a fastigiated to dendritic-shaped periphery. Microscopy of the colony in lactophenol blue mount demonstrated large fusiform thick-walled macroconidia with 1–6 septa. Some of them were characteristic incurved macroconidia.

The anthropophilic species are mainly represented compared to zoophilic species. Indeed, 86% of the characterised dermatophytes of tinea capitis were anthropophilic (*p*-value > 0.0001). No significant relation can be established between the majority of dermatophytes species and the origin of the patient. Only one statistically significant relation can be made for *T. soudanense*. Indeed among *T. soudanense* with known ethnic origin, 15/25 (60.2%, *p*-value = 0.003) cases were from Guinean origin. Among the different age ranges, we can see that among the age range of 0–4 years, *M. audouinii* is predominant (*p*-value = 0.057), and among the age range 5–9 years, *T. soudanense* is the main agent (*p* = 0.049). Regarding the geographical areas in Belgium, the proportion of *T. tonsurans* and *T. soudanense* is more frequent in Brussels compared to the other regions (*p*-value = 0.04 and *p*-value > 0.0001, respectively). On the contrary, the proportion of *M. audouinii* and *M. canis* is smaller in Brussels, and the agents are more predominantly found in other regions of Belgium (*p*-value = 0.016 and *p*-value = 0.019, respectively). *M. audouinii* accounted for 46.6% of the cases of tinea capitis in Liege, so it was predominant in this area. We asked the patients to mention if other family members were also affected by tinea capitis. Among the received answers, and confirmed by laboratory diagnostic attesting the presence of dermatophyte infection (*n* = 126), 105 (83.3%) had a member of the family affected by tinea capitis (*p*-value < 0.0001) (these data do not consider possible asymptomatic carriage among family if they were not screened for dermatophytes). We also asked if recent history of travel abroad was observed in subjects with tinea capitis and 61 (60.4%, *n* = 101) mentioned recent travel abroad (*p*-value = 0.03). Holding pets did not predispose to tinea capitis (*p*-value < 0.0001) as the majority of the patients did not have any pets (71/81, 87.7% among reported cases).

### 3.2. MIC Determination and Screening for Mutation in SQLE Gene among T. mentagrophytes

**Figure 4:**
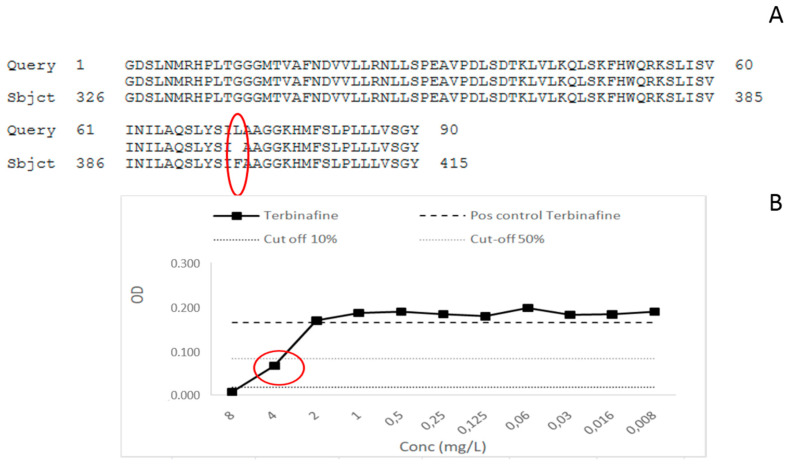
(**A**) Mutation Phe397Leu on the SQLE gene of *T. mentagrophytes* strain n° 13-180730-0023 surrounded in red. (**B**) MIC determination for terbinafine; the MIC value is surrounded in red and is equal to 4 mg/L.

The five strains of *T. mentagrophytes* characterised in this paper were screened for the squalene epoxidase (SQLE) mutation as it has been demonstrated that zoophilic strains of *T. mentagrophytes* could present some resistance to terbinafine, which is the main treatment of tinea capitis in Belgium [18,19]. The results of MIC determination by broth microdilution method have demonstrated for one of the five strains an MIC for terbinafine of 4 mg/L. For this strain, MIC for itraconazole was 0.016 mg/L, 0.5 mg/L for voriconazole, and 0.06 mg/L for amorolfine. Thus, this strain only showed a resistance to terbinafine as sensitive strains are described to have MIC lower than 0.25 mg/L for this antifungal [16,17]. The other four *T. mentagrophytes* strains showed no resistance to any antifungal tested and presented MIC lower or equal to 0.03 mg/L for terbinafine (Table 3). The SQLE gene was then screened for mutations as described by Yamada et al. [17]. After sequencing and analysis, one mutation, Phe397Leu, was observed in one of the five screened strains, the same one that showed an MIC for terbinafine of 4 mg/L (Figure 4 for illustration). The patient was a 25 year old man, he presented itchy lesions all over the inguinal region and the trunk that spread for several weeks. Some lesions were squamous, with some being annular on the trunk. The patient also presented squamous lesions on the occiput. The patient had no animals and did not mention any travel in India. His sister was also affected by a dermatophyte infection. He received oral terbinafine (250 mg/day) for 3 weeks, associated with sulconazole nitrate (10 mg/mL) and ketoconazole shampoo. The patient never came back to the consultation, so we do not know if the lesions were healed. The other four strains of *T. mentagrophytes* shared a wild-type profile regarding SQLE gene.

## 4. Discussion

Our study highlighted the current tendencies in tinea capitis epidemiology in Belgium. Males are more often affected by tinea capitis than females in Belgium. Among children, this observation has already been shown all around Europe [20,21,22,23]. Similar to previous reports from several European countries, patients suffering from tinea capitis were mainly patients of African origin (81% of informed cases), where species like *M. audouinii, T. soudanense,* and *T. violaceum* are endemic [24,25,26,27,28]. However, few travels to African countries were reported in this study, showing that infections are mainly acquired in Belgium. As has been mainly reported [20,29,30,31], children are more affected by tinea capitis than adults, as children younger than 10 represent 81% of the recorded infections. Regarding the prevalence of dermatophytes causing tinea capitis in Belgium, our study shows that *M. audouinii* is the first aetiological agent responsible for tinea capitis, followed by other anthropophilic species such as *T. soudanense* and *T. tonsurans*. Zoophilic species such as *M. canis, T. benhamiae* or *T. mentagrophytes* are present, but few are represented. This tendency has also been seen in other European countries. In western Europe, *M. audouinii* is present in Switzerland, where it has been responsible for several outbreaks in schools [32]. This species has also been described to be responsible for tinea capitis outbreaks in Germany, mainly imported from children after vacations in Africa that then spread to other children in communities or their families [2]. In France, *M. audouinii* is present but not the first agent of tinea capitis [8]. In Switzerland and in Italy, *T. violaceum* has been reported in many cases of tinea capitis [20,33,34]. In our study, we only have a few *T. violaceum* recorded. This can be explained by different immigration waves among the considered countries as *T. violaceum* is endemic in North and East Africa [35,36], while *M. audouinii* and *T. soudanense* are highly present in western Africa. Many Belgian migrants are from Western and Central Africa, but this is different in other countries. Indeed, when we consider the country of origin for recorded cases in our study, the majority come from western African countries such as Guinea, Cameroun, Ivory Coast, and Angola. Eastern African countries, such as Ethiopia, where we find *T. violaceum,* are not represented among our cases, which can explain the small rate of *T. violaceum* isolated in this study. A recent study conducted in the urban area of Paris has shown that *T. tonsurans* was mainly responsible for tinea capitis in France, as is also the case also in United Kingdom [9,14,37]. Systemic griseofulvin is the indicated treatment for tinea capitis and is licensed in some countries such as France, but in other countries, it is not, such as Belgium and Switzerland [20,38]. This difference in the treatment of tinea capitis can explain the large rate of *M. audouinii* seen in our study and in other European countries such as Switzerland, where griseofulvin is not used. On the contrary, in France, *M. audouinii* is not the first aetiological agent of tinea capitis, even though this species is also circulating there [8,9]. Indeed, it has been already described in a meta-analysis of randomized clinical trials that oral terbinafine was less efficient in *Microsporum* species than in *Trichophyton* species, while griseofulvin showed similar activities in both genera, which can explain the different number primary aetiological agents for tinea capitis between two neighboring countries [39]. Previous data from Belgium are quite consistent with our results. However, we can notice that while anthropophilic strains are still predominant, with *M. audouinii* and *T. soudanense* being the two most prevalent aetiological agents of tinea capitis, the profile of dermatophytes giving rise to tinea capitis in Belgium has still been changing for the last two decades. Indeed, the study done by Kolivras et al. in Brussels in 2002 showed that *Microsporum audouinii* was responsible for 39.34% of tinea capitis followed by *T. soudanense* (28.69%), then *T. violaceum* (18.03%) and *T. tonsurans* (3.28%) [40]. Our study demonstrated a higher rate of *T. tonsurans* (16.9%) and a lower rate of *T. violaceum* (8.3%). Again, this can be explained by the changing profiles of immigration waves in Belgium during the two last decades. In both studies, few zoophilic strains are represented. This study has limitations, and despite the fact that it was a prospective study, the epidemiological record of information about the patients was not easy. Indeed, dermatologists did not participate willingly, and they did not fill the form with the patient for every case of tinea capitis they encountered, as requested by the National Reference Center for Mycoses. Therefore, much epidemiological information is unfortunately missing, but some tendencies can clearly be observed nonetheless.

Our study highlighted the detection of *N. incurvata* as a new agent of tinea capitis in Belgium. This geophilic species has never been reported to be responsible for tinea capitis in Belgium before. The complex of *Nannizzia gypsea* is known to encompass three geophilic species, including *N. gypsea*, *N. incurvata,* and *N. fulva* [41,42,43]. *N. incurvata* is a rare or possibly underdiagnosed geophilic dermatophyte. Discriminating *N. incurvata, N. gypsea*, and *N. fulva* from the other forms of macroconidia has certain difficulties, which is why doing ITS–PCR is mandatory to distinguish this species from others of the same clade. Some cases of kerion due to *N. incurvata* have already been described in Japan, Sri Lanka, Vietnam, and Cambodia, but this is the first documented case in western Europe [44,45]. One patient had just travelled to Panama, but until now, no reported cases of tinea capitis due to *N. incurvata* are reported in this country.

Among the five strains of *T. mentagrophytes* isolated in this study, one of them showed a resistance profile to terbinafine confirmed by the mutation Phe397Leu highlighted by SQLE gene sequencing and already described to be responsible for terbinafine resistance in *T. rubrum* and *T. mentagrophytes* [17,46]. All around the world, in India, Japan, and Europe, the emergence of dermatophyte resistance to terbinafine is described, essentially caused by the *T. mentagrophytes* type VIII frequently having mutations at positions Leu393Phe, Phe397Leu of the squalene epoxydase gene [47]. Other point mutations in the SQLE gene inducing terbinafine resistance have been described in Europe in *Trichophytons,* such as Phe415Ile/Ser/Val and His440Tyr [17]. In a recent study, Saunte et al. identified L393S, H440Y/F484Y, and I121M/V237I substitutions responsible for low to moderate terbinafine resistance in *Trichophyton rubrum* and *T. interdigitale* [18]. In Iran, five isolates with terbinafine MICs ≥32 µg/mL were recently described. They belonged to *T. mentagrophytes* type VIII and harbored two alternative *SQLE* gene sequence variants, leading to Phe397Leu and Ala448Thr or Leu393Ser and Ala448Thr substitutions in the enzyme [48]. A new mutation in the SQLE gene of *T. mentagrophytes* associated with terbinafine resistance in a couple with disseminated tinea corporis has also been described recently. This concerned an A1223T point mutation leading to a Q408L substitution identified by DNA sequencing of the SQLE gene of the isolate [49]. These mutations in the SQLE gene can lead to epidemics or extensive infections due to the dermatophyte [18,19,50,51]. Indeed, in our study, the identified *T. mentagrophytes* resistant strain gave rise to tinea capitis lesions but also a persistent extended tinea corporis with originating on inguinal folds. Recently, resistance mechanisms to terbinafine in *M. canis* isolated from a cat have also been described [52,53]. *T. mentagrophytes* strains resistant to terbinafine were also newly isolated from asymptomatic foxes in Poland. In the genomes of all resistant strains exhibiting elevated MICs to terbinafine (16 to 32 µg/mL), single-point mutations leading to Leu393Phe substitution in the squalene epoxidase enzyme were revealed. [54]. This confirms the need to be careful regarding the emergence of dermatophytes resistant to terbinafine that occur not only in humans but also in animals, which can rapidly give rise to extended outbreaks. Squalene epoxidase substitutions between Leu393 and Ser443 could serve as markers of resistance in the future, as suggested by a recent paper from a multicenter study conducted in India [55]. In this recent paper, two novel substitutions in resistant *Trichophyton* strains, Ser395Pro and Ser443Pro, were discovered. Thus the finding of a *T. mentagrophytes* strain, potentially of type VIII (to be confirmed by genotypical analysis), among our collected strains during the national study on tinea capitis demonstrates the need to screen this type of resistance among Belgian strains as it can rapidly give rise to outbreaks, as is the case in India, or extensive tinea infections. We advise close monitoring of resistance, with antifungal susceptibility tests done more systematically in dermatophytoses that do not respond to conventional treatment as terbinafine resistant strains are likely to spread worldwide. The molecular characterization of the mutation should be restricted to recalcitrant cases or to strains that are growing by a screening of resistance in culture as described by Yamada et al., in which a large-scale culture screening of *T. rubrum/mentagrophytes* strains is done on a medium containing 0.2 mg/L of terbinafine before SQLE gene characterization by PCR [17]. Clinical laboratories could use this method of screening or an equivalent method for all *T. rubrum*/*T mentagrophytes* (or strains responsible for relapses or recalcitrant dermatophytoses) found by culture to better characterize resistance mechanisms in dermatophytes. Guidelines on tinea capitis management should be adapted regarding this emerging resistance phenomenon. British guidelines from 2014 do not mention this relatively new observation [56], and the recent German published guidelines do not focus enough attention on this new phenomenon of resistance among dermatophytes even though this phenomenon has been well described since 2017 [57]. In Belgium, there are no recent published guidelines that mention terbinafine resistance for the management of recalcitrant tinea capitis, the last one being from 2004, when nothing about the molecular characterization of the SQLE resistance was yet known [58].

## Figures and Tables

**Table 1:** Distribution of the 337 cases of tinea capitis by laboratory, sex, age, and nationality.

Variable	Categories	N	Number (Percent)
Laboratory		337	
	CHR Citadelle Liège		15 (4.5)
	CHU de Liège		52 (15,4)
	CHU—UCL Namur		2 (0.6)
	CHU St Pierre BXL		245 (72.7)
	Clinique St Luc de Bouge		7 (2.1)
	Vivalia Bastogne		1 (0.3)
	CEBIODI Bruxelles		6 (1.8)
	UZ Leuven		3 (0.9)
	Laboratoire Luc Olivier		1 (0.3)
	CHVE		3 (0.9)
	Clinique Montlegia Liege		1 (0.3)
	Universitair Ziekenhuis Gent		1 (0.3)
Sex		337	
	Woman		123 (36.5)
	Man		214 (63.5)
Age (Years)		337	
	0–4		110 (32.6)
	5–9		165 (49.0)
	≥10		62 (18.4)
Nationality		104	
	Non-African		85 (81.7)
	African		19 (18.3)

**Table 2:** Distribution of the 337 cases of tinea capitis by country of origin, history of travels, familial infections, infections acquired in schools, or animal holding.

Variable	Categories	N	Number (Percent)
Country of origin		119	
	Algeria		1 (0.8)
	Angola		7 (5.9)
	Belgium		4 (3.4)
	Cameroon		14 (11.8)
	Congo		14 (11.8)
	Ivory Coast		10 (8.4)
	Guinea		26 (21.8)
	Morocco		11 (9.2)
	Turkey		2 (1.7)
	Senegal		9 (7.6)
	Ukraine		1 (0.8)
	Romania		1 (0.8)
	Togo		3 (2.5)
	Benin		2 (1.7)
	Rwanda		3 (2.5)
	Italy		1 (0.8)
	Mauritania		1 (0.8)
	Sierra Leone		3 (2.5)
	Niger		5 (4.2)
	Somalia		1 (0.8)
History of travels		101	
	No		40 (39.6)
	Yes		61 (60.4)
Familial infection		126	
	No		21 (16.7)
	Yes		105 (83.3)
Infection acquired in school		18	
	No		11 (61.1)
	Yes		7 (38.9)
Animal holding		81	
	No		71 (87.7)
	Yes		10 (12.3)

**Table 3:** Minimal inhibitory concentrations (MIC) against four antifungals and SQLE profile of the 5 strains of *T. mentagrophytes* isolated in the study. TER: terbinafine, VOR: voriconazole, ITRA: itraconazole, AMOR: amorolfine.

Strain N°	MIC TER (mg/L)	MIC VOR (mg/L)	MIC ITRA (mg/L)	MIC AMOR (mg/L)	SQLE Mutation
*T. mentagrophytes* 180730-0023	4	0.5	0.016	0.06	Phe397Leu
*T. mentagrophytes* 180814-0035	0.03	0.5	0.5	0.5	/
*T. mentagrophytes* 180925-0031	0.03	0.5	0.125	0.5	/
*T. mentagrophytes* 181106-0067	0.03	0.5	0.03	0.125	/
*T. mentagrophytes* 181114-0030	0.016	0.125	0.125	0.125	/
